# Oviposition behaviour and emergence through time of the small blue butterfly (*Cupido minimus*) in a nature reserve in Bedfordshire, UK

**DOI:** 10.1007/s10841-021-00360-5

**Published:** 2021-12-06

**Authors:** Esme Ashe-Jepson, Andrew J. Bladon, Greg Herbert, Gwen E. Hitchcock, Richard Knock, Colin B. H. Lucas, Sarah H. Luke, Edgar C. Turner

**Affiliations:** 1 Department of Zoology, University of Cambridge, Downing Street, Cambridge, CB2 3EJ UK; 2 Bedfordshire and Northamptonshire branch of Butterfly Conservation, Registered office: Manor Yard, East Lulworth, Wareham, Dorset, BH20 5QP UK; 3 The Wildlife Trust for Bedfordshire, Cambridgeshire and Northamptonshire, The Manor House, Broad Street, Cambourne, Cambridge, CB23 6DH UK; 4 49 Mill Road, Beccles, Suffolk, NR34 9UT UK

**Keywords:** Abundance, Butterflies, Climate change, Conservation management, Ecology, Temperature

## Abstract

**Abstract:**

Climate change affects butterflies in many ways, influencing the timing of emergence and reproduction, habitat preferences, and behaviour. The small blue (*Cupido minimus* Fuessley, 1775) is highly specialised in its host plant requirements, feeding on the seeds of a single species, kidney vetch (*Anthyllis vulneraria*), on which the larvae occur singly to avoid cannibalism. The butterfly is likely to be vulnerable to temperature-related changes in oviposition, adult emergence, and host plant flowering times, and is, therefore, a good model species for investigating climate change-related impacts. Using 26 years of data from the national UK Butterfly Monitoring Scheme (1993–2019) from one nature reserve, and 4 years of targeted egg searches (2006, 2007, 2008, 2020) from three reserves in Bedfordshire, UK, we investigated the effects of local temperature on small blue emergence date and total abundance, whether flowerhead or local environmental characteristics predicted small blue oviposition behaviour, and whether this changed between years. Small blue adults emerged on earlier dates over time, and earlier in years with higher maximum February temperatures. Total adult abundance was not predicted by monthly temperatures or total abundance in the previous year. Oviposition behaviour was broadly consistent across years, with egg presence more likely and egg abundance higher on kidney vetch flowerheads that were taller than the surrounding vegetation, and surrounded by taller vegetation and fewer mature flowerheads. The effect of solar radiation differed between years, with a negative effect on the probability of egg presence in 2007 and 2008, but a positive effect in 2020. Egg abundance per flowerhead was highly variable between years, with 2006 having four times more eggs per flowerhead than other years. This was likely driven by high adult abundance in 2006, which could have increased competition for flowerheads.

**Implications for insect conservation:**

Our results indicate that management for greater availability of taller kidney vetch amongst taller vegetation would encourage small blue oviposition on a greater number of flowerheads, providing a possible means of reducing competition and increasing larval survival, and that this would be effective despite variation in adult abundance between years. The high level of competition we observed in the year with the highest adult abundance indicates that higher numbers of host plants should be encouraged to reduce competition and larval cannibalism in peak years, increasing the likelihood of long-term population persistence and growth.

**Supplementary Information:**

The online version contains supplementary material available at 10.1007/s10841-021-00360-5.

## Introduction

Climate change is a major threat facing wild populations (Thomas et al. 2004), with predicted impacts in temperate regions including increasing summer (Battisti and Naylor 2009) and winter (Kreyling 2010) temperatures, changes to precipitation (Trenberth 2011), and increased frequency and intensity of extreme events (IPCC 2014). The effects of climate change have been detected in many taxa, particularly insects (Elias 1991; Kingsolver et al. 2011). Temperature affects insect physiology, behaviour, survival, growth, and development, causes disruption to the synchrony of insect-plant interactions, induces poleward range shifts, and drives changes in populations of natural enemies (Elias 1991; Bale et al. 2002). In butterflies, temperature has been known to influence emergence time (Roy and Sparks 2000), habitat preference (Davies et al. 2006; Ashton et al. 2009), behaviour (Cormont et al. 2011), and distribution (Hill et al. 2002).

To understand, predict, and manage the impacts of climate change, knowledge of a species’ status and change in demography over time is needed. Time-series measures of phenology, such as emergence date, are used to track and predict long-term change (Forister and Shapiro 2003; Stefanescu et al. 2003), and are vital for uncovering changes in species interactions, which can be driven by temperature variation (Roy and Sparks 2000). Another relevant, but understudied, aspect of butterfly biology is behaviour (Dover 1997; Kallioniemi et al. 2014), including oviposition choice. Oviposition behaviour is important for butterfly survival and reproduction, and can be integrated into effective conservation of endangered and declining species (Konvička and Kuras 1999; Bergström 2005; Turner et al. 2009), by maintaining host plants in the most favourable conditions for larvae. Like many holometabolous insects, butterfly larvae are less mobile than adults (Hagstrum and Subramanyam 2010), so selection of a suitable host plant by females during oviposition can determine the fitness and survival of offspring (Bergman 1999).

Oviposition preferences vary between species and individuals, and in direction and strength between years. For example, orange-tips (*Anthocharis cardomines*) and Edith’s checkerspots (*Euphydryas editha*) make oviposition decisions based on aspects of plant structure (Courtney 1982; Parmesan 1991), whilst silver-spotted skippers (*Hesperia comma*) and black-veined whites (*Aporia crataegi*) make egg-laying decisions based on temperature (Davies et al. 2006; Merrill et al. 2008). Intraspecific differences can be affected by female larval experience (Cahenzli et al. 2015), adult experience (Rausher 1978), and genetic variation (Wiklund 1974; Tabashnik et al. 1981).

The small blue (*Cupido minimus*) is a monophagous Palearctic Lycaenid butterfly, and a specialist of calcareous grasslands (Asher et al. 2001). Although widespread across Europe and temperate Asia, it is locally restricted in some regions, and has undergone declines in distribution Europe in recent years (Asher et al. 2001; van Swaay et al. 2010). In the UK, the species is widespread but rare and declining in distribution, with a 44% decline in distribution from 1976 to 2014 (Fox et al. 2015). The small blue has high inter-annual variation in abundance at the local and national scale (Botham et al. 2019), with characteristic peak years followed by low abundance years. This can leave small and isolated populations vulnerable to extinction (Bourn and Warren 2000).

Small blues are predominately bivoltine in the UK (univoltine in Scotland), with adults flying from mid-May–June and late July–August (Asher et al. 2001). The only larval host plant used is kidney vetch (*Anthyllis vulneraria*), a perennial that flowers from June to September (Charman et al. 2009; Lorenz et al. 2020). Females lay eggs singly between the florets of developing flowerheads (Morton 1985) and leave chemical cues thought to deter other females from laying (Thomas and Lewington 2016). This is likely to be because caterpillars are cannibalistic, and typically only a single caterpillar on each flowerhead will survive (Asher et al. 2001). However, multiple eggs are often found on the same flowerhead, suggesting that deterrents may be ineffective, or that oviposition sites are limited. Larvae feed on the developing seeds and anthers, requiring a close phenological match with their host plant (Peterson 1997). Fully grown fourth instar larvae either overwinter at the soil level, or pupate and emerge as part of a second brood. After overwintering, larvae do not resume feeding, and pupate in spring at ground level. Adults emerge after approximately 2 weeks (Eeles 2019).

The small blue may be particularly vulnerable to climate change, due to its temperature sensitivity, specific reproductive requirements, patchy distribution, and limited dispersal. Adults emerge earlier in warmer springs (Roy et al. 2015; Macgregor et al. 2019), putting them at risk of phenology mismatch with host plants if kidney vetch do not respond in the same way (Macgregor et al. 2019). The butterfly’s limited dispersal (Cowley et al. 2001), geographical isolation (Asher et al. 2001), and habitat specialisation (van Swaay 2002) may also limit its ability to expand north in response to climate change (Hill et al. 2002). Roy et al. (2015) quantified the effects of temperature on adult emergence using 3-month running means, but there has been little investigation of how temperature affects abundance, and no study has quantified oviposition behaviour across years. Investigating these traits in parallel may provide important insights into the ecology of this species, and a valuable case study for developing understanding of the links between the effects of temperature and other inter-annual differences on oviposition.

Here, we combine long-term butterfly transect data and data on oviposition from three nature reserves in Bedfordshire, UK, to investigate how small blue adult abundance and emergence times are influenced by local temperature change, and whether factors predicting oviposition vary between years. We address the following questions:

1. Are annual first emergence date or total abundance of small blues at a local scale affected by temperature, and have these changed over time?
2. Is small blue oviposition behaviour influenced by flowerhead or local environmental characteristics, and does this differ between years?

## Methods

### Study sites

**Fig. 1:**
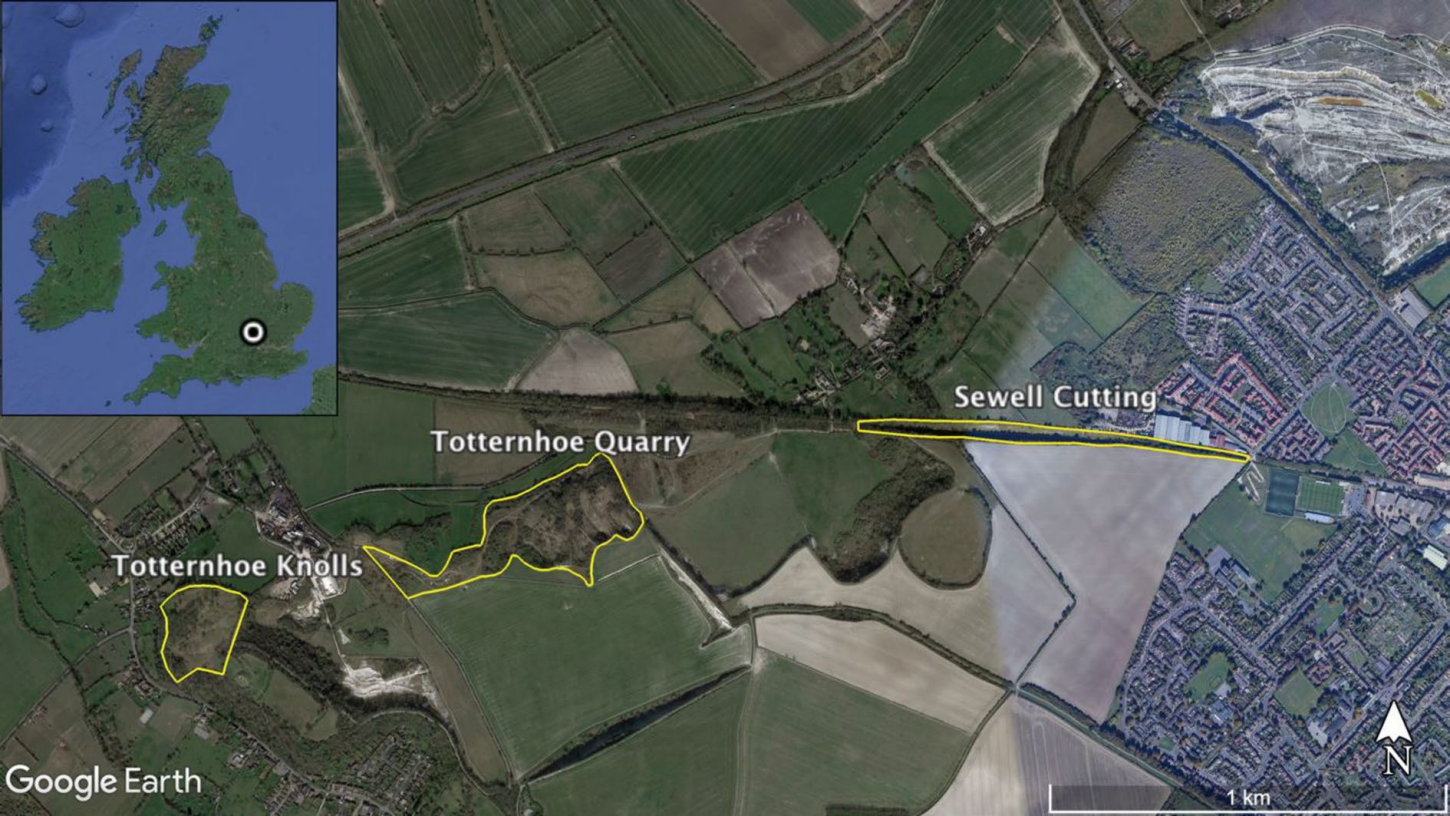
Study site locations in the UK (inset), and in Bedfordshire. The three sites (Totternhoe Quarry, Totternhoe Knolls and Sewell Cutting) are outlined in yellow. Source: Google Earth Pro v.7.3.3.7786, 51°53′40.62″ N, 0°33′37.46″ W, eye alt 4.28 km. Data SIO, NOAA, US Navy, NGA, GEBCO. Image Landsat/Copernicus. © Google Earth. Imagery date: 25 March 2020 (accessed 14 January 2021)

Data collection took place in Totternhoe Quarry [51.884410, − 0.571340], Sewell Cutting [51.8944252, − 0.5522611] and Totternhoe Knolls [51.889757, − 0.580237] nature reserves, near Dunstable, Bedfordshire, UK (Fig. 1). Totternhoe Quarry is an 8.9-ha Site of Special Scientific Interest (SSSI) and former chalk quarry containing high quality chalk grassland, with areas of short and long grass, scrub, and highly variable topography. Sewell Cutting is a 3.6-ha disused railway cutting with steep chalk cliffs. Totternhoe Knolls is a 4.0-ha SSSI, similar to Totternhoe Quarry, with variable vegetation and topography. All three reserves are managed by the Wildlife Trust for Bedfordshire, Cambridgeshire and Northamptonshire (BCNWT), and are important sites for manyPinvertebrates, including butterflies such as the small blue, the chalkhill blue (*Polyommatus coridon*), and the Duke of Burgundy (*Hamearis lucina*).

### Adult abundance and monthly temperature data

Weekly counts of small blues from Totternhoe Quarry were obtained from the UK Butterfly Monitoring Scheme (BMS), accessed through the Bedfordshire Biological Records Centre (all data collected by GH). The methods are described in detail by Pollard and Yates (1993), and summarised briefly here. A fixed transect route of 2.12 km, running through representative habitats across the reserve, was walked weekly from April to September each year, provided weather conditions met set criteria: (1) between 13 and 17 °C and sunny or (2) above 17 °C in any conditions except for rain, and (3) wind speeds below 5 on the Beaufort scale. All small blues seen within 2.5 m either side of the transect, and up to 5 m ahead of the observer, were recorded. Data were available in all years from 1993 to 2019, except 2017. From these weekly counts, the date of first emergence (calendar date when the first small blue was seen) and the total abundance of the first generation (the sum of all individuals seen before mid-July) were calculated for each year.

Minimum and maximum daily temperatures from January to June were obtained from the Met Office MIDAS dataset from a nearby weather station in Woburn [52.01400, − 0.59457] (Met Office 2021) (except for 2000 when this weather station was not operational). These months were selected because temperatures in the months of rapid larval and pupal development prior to the flight period have been found to influence butterfly phenology for a range of species (Stefanescu et al. 2003), including small blue (Roy et al. 2015). However, as there was uncertainty in what months were most important, a range were included, but limited to 6 months to avoid overfitting models. Daily minimum and maximum temperatures were averaged for each calendar month to produce monthly values.

### Egg searches, and host plant and environmental characteristics

Data on egg location and host plant and environmental characteristics were collected in 2006, 2007, 2008, and 2020, during the early summer flight season and first generation of small blue. There were differences in the scale of searches across years, but general methods were consistent.

#### 2006

All searches were conducted on 12th June at Totternhoe Quarry. A pragmatic approach was taken, with hand-searches of as many kidney vetch flowerheads as possible completed in areas known to be used by small blues. For flowerheads on which eggs were found (Fig A1), the number of eggs per flowerhead and the surrounding environmental characteristics were recorded. Flower height was recorded by measuring from the ground to the tip of the flowerhead along the stem. Vegetation height was recorded by gently resting an A4 board flat on the surrounding vegetation, to exclude any long single stems, before measuring the vegetation height as the distance from the ground to the board. This method is similar to the ‘drop disc’ method, discussed by Stewart et al. (2001).

#### 2007

All searches were conducted in mid to late June at Totternhoe Quarry, with all flowerheads within areas known to be used by small blues searched. For each flowerhead (including those with no eggs) we recorded: flower and vegetation height, slope (estimated visually in degrees), aspect (bearing that the slope was facing, measured with a compass), number of mature flowerheads within a 30-cm radius (referred to hereafter as Mature), and number of developing flowerheads within a 30-cm radius (hereafter: Buds).

#### 2008

All field work was conducted between mid-June and mid-July, with all flowerheads recorded as in 2007. Data collection took place at Totternhoe Quarry, Totternhoe Knolls, Sewell Cutting and in surrounding land. Within all kidney vetch patches, a maximum of 50 flowerheads were selected randomly and searched for eggs. All previously recorded variables, except Mature and Buds, were measured towards the end of the survey period.

#### 2020

All fieldwork was conducted in July at Totternhoe Quarry. Surveys were later than other years due to COVID-19. However, surveys were still possible as flowerheads were intact and empty eggs were present. Within all kidney vetch patches, a maximum of 10 flowerheads were selected randomly and searched for eggs. All previously recorded variables, including Mature and Buds, were measured.

### Data processing and statistical analyses

Analyses were performed in R version 3.6.1 (R Core Development Team, http://www.r-project.org). Data dispersion for Poisson regressions was checked using the ‘AER’ package (Kleiber and Zeileis 2008). Where data were under-dispersed, Conway-Maxwell-Poisson regressions were fitted using the ‘spaMM’ package. Where data were over-dispersed, negative binomial regressions were fitted using the ‘MASS’ package (Venables and Ripley 2002). Assumptions of the chosen error structure were tested before model fitting. Akaike Information Criterion (AIC) values and AIC corrected for small sample size (AIC_c_) were examined using the ‘MuMIn’ package (Barton 2020). Type II analysis-of-variance tables for models were examined using the ‘car’ package (Fox and Weisberg 2019).

Flower apparency was calculated as the difference between flower height and vegetation height. The ‘DirectRadiation’ function in the ‘solrad’ R package was used to calculate solar direct beam radiation on a surface (in W/m^2^) (Seyednasrollah 2018) (hereafter: solar radiation). Solar radiation at each flower location was calculated using longitude, latitude, elevation, slope, aspect, day of the year and time. As solar radiation varies throughout the year and with time of day (Rorison et al. 1986), four values were calculated for each kidney vetch location at midday in the middle of each season (mid-spring, Julian day 105; mid-summer, Julian day 196, mid-autumn, Julian day 288; mid-winter, Julian day 15) and averaged to produce mean solar radiation. This was included as an explanatory variable, combining slope and aspect into a single biologically-relevant metric.

### Temperature and demographic trends

Explanatory variables (mean monthly maximum and minimum temperature, and year) were tested for collinearity, and variables with pairwise correlation coefficients > 0.7 were excluded (Dormann et al. 2013). Mean monthly minimum temperature was excluded in order to retain mean monthly maximum temperature, which is more likely to affect larval development and survival (Dell et al. 2005), and under climate change unseasonal maximum temperatures are becoming more common (Lobell et al. 2007). There was no significant correlation between year and any temperature variable. Inspection of Auto- and Cross- Covariance and Correlation Function (ACF) plots of residuals did not show evidence of temporal autocorrelation.

To test whether temperature predicted small blue emergence date, a linear regression with a Gaussian error distribution was fitted, with day of first emergence as the response variable and mean monthly maximum temperatures from January to June and year as explanatory variables. Year was included as a fixed effect to test for changes in emergence over time. No interaction terms were included. To test whether temperature predicted total abundance, a linear regression with a negative binomial error distribution was fitted, with total abundance as the response variable and mean monthly maximum temperatures from January to June, year, and total abundance in the previous year as explanatory variables. No interaction terms were included. AIC_c_ was used to select the best model. If the difference in AIC_c_ between models was > 2, the model with the smallest AIC_c_ was selected. Where the difference was < 2, the simplest model was selected (Burnham and Anderson 2010).

### Oviposition behaviour: egg presence

Oviposition behaviour analyses were split between egg presence and abundance, to evaluate different research questions. The egg presence analysis aims to identify traits that are attractive to egg-laying females, and therefore desirable goals for reserve management. The egg abundance analysis aims to identify traits which result in multiple eggs on a single flowerhead, increasing competition and larval cannibalism, and are therefore undesirable for management.

Explanatory variables (flower height, vegetation height, flower apparency, mean solar radiation, Mature, Buds) were assessed for collinearity, using the methods outlined above. Two pairs of variables were strongly correlated: flower height and apparency (correlation coefficient = 0.78), and flower height and vegetation height (correlation coefficient = 0.70). Therefore, flower height was excluded from analyses to retain vegetation height and apparency, which were not strongly correlated (correlation coefficient = 0.10).

To test whether environmental characteristics predicted egg presence, and whether this changed over time, a logistic regression was fitted with egg presence as the binary response variable, and flower apparency, vegetation height, mean solar radiation, and year as explanatory variables. The two-way interactions of each explanatory variable with year were included to test for inter-annual variation in the response of oviposition to flowerhead or environmental characteristics. AIC values were again used to select the best model. Because flowerheads without eggs were not recorded in 2006, data from this year were excluded from this analysis.

To test the effect of environmental characteristics which were only collected in 2007 and 2020, an additional set of models were fitted using data from these 2 years. These models included Mature, Buds and the two-way interactions of these terms with year as additional explanatory variables. AIC was used to select the best model.

### Oviposition behaviour: egg abundance

To test whether the abundance of eggs on a flowerhead was predicted by environmental characteristics, and whether this changed over time, a Conway-Maxwell-Poisson (COMPoisson) regression with a log-lambda link was fitted, as the data were under-dispersed (Huang 2020). Abundance per flowerhead was fitted as the response variable, and flower apparency, vegetation height, year, and each two-way interaction including year as explanatory variables. Due to the high number of flowerheads with no eggs, and because we had tested for environmental predictors of egg presence, zero counts were excluded. Mean solar radiation was excluded from this analysis because slope and aspect were not recorded in 2006. AIC was used to select the best model.

To investigate the effect of environmental characteristics recorded in 2007 and 2020 only, an additional set of models was fitted using data from these years. Mean solar radiation, Mature, and Buds were fitted as additional explanatory variables, and two-way interactions between these variables and year. AIC was used to select the best model.

## Results

### Temperature and demographic trends

Across the 26 years, small blue first emergence ranged from 13th May to 13th June (day 133− 165) (Fig A2). Annual total abundance ranged from 2 to 38 adults (mean: 17.2) (Fig A3). Mean monthly maximum temperatures ranged from: 2.6− 9.4 °C in January, 3.3−10.9 °C in February, 4.2−13.3 °C in March, 10.1−15.2 °C in April, 13.3− 17.7 °C in May, and 16.4−21.0 °C in June (Figure A4).

### Emergence date

**Fig. 2:**
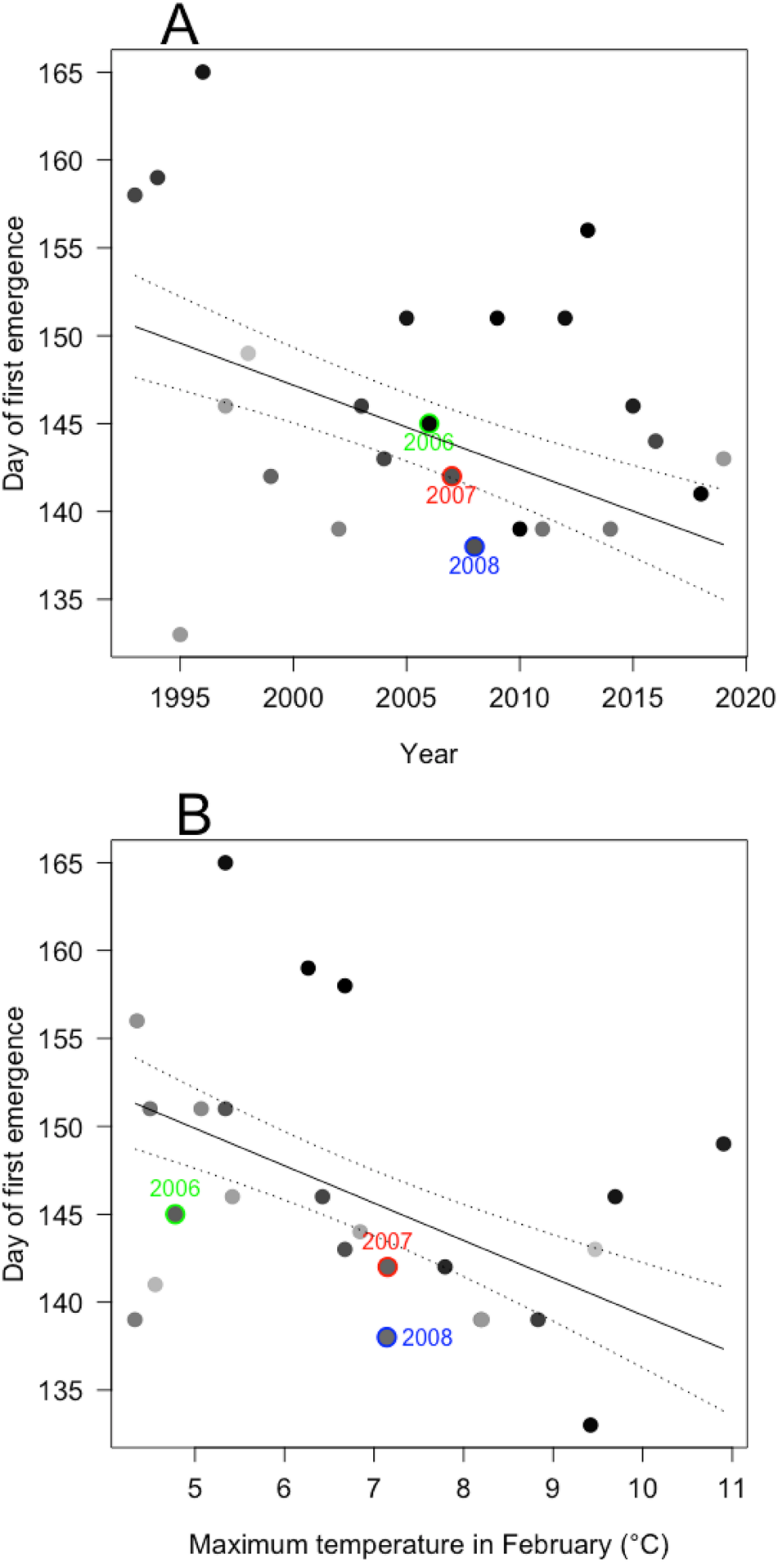
The day of first small blue emergence at Totternhoe Quarry reserve over time (**A**) and compared to average maximum temperature in February (**B**). Points represent individual years, and are shaded on a continuous scale by (**A**) average maximum temperature in February and (**B**) year, where black = low value/earlier year, light grey = high value/later year. Black lines show the predicted values from the multiple regression with the lowest AIC_c_ value, with maximum temperature in February (**A**) and year (**B**) held at their mean. Dashed lines show standard errors. Years when oviposition behaviour were recorded are highlighted: 2006 (green), 2007 (red), and 2008 (blue). (Color figure online)

The optimal model included year and maximum temperature in February (Table A1). Small blues emerged earlier in more recent years (F = 7.294, d.f. = 1, p = 0.013; Fig. 2A), and following higher average maximum temperatures in February (F = 8.397, d.f. = 1, p = 0.009; Fig. 2B). For each passing year, small blue emergence advanced by 0.48 days on average, equating to 12.5 days over 26 years. For every 1 °C increase in mean maximum temperature in February, emergence was on average 2.13 days earlier.

Of our three focal years, 2008 had the earliest first emergence, followed by 2007 then 2006 (day 138, 142 and 145, respectively), and all focal years were earlier than average (x̄ = 146). 2006 had a lower mean maximum temperature in February than 2007 and 2008 (4.80 °C, 7.15 °C and 7.14 °C, respectively). February 2006 was one of the coldest across our study, whereas 2007 and 2008 were warmer than average (x̄ = 6.80 °C).

### Total abundance

The optimal model for total abundance was the null model (Table A2). Mean monthly maximum temperature, year, and total abundance in the previous year did not predict total abundance. Of our three focal years, 2006 had a higher total abundance than 2007 or 2008 (26, 4 and 8 individuals, respectively), and was above average compared to all years (x̄ = 17.2). Total abundance in 2007 was among the lowest across the 26 years.

### Oviposition behaviour

In total, 4265 kidney vetch flowerheads were searched for eggs, with 1237 eggs found on 660 flowerheads (Fig A5). In 2006, a total of 471 eggs were recorded on 83 flowerheads. In 2007, from 2,016 searched flowerheads, 360 eggs were recorded on 262 flowerheads (13% of flowerheads with eggs). In 2008, from 1,507 searched flowerheads, 353 eggs were recorded on 270 flowerheads (18% of flowerheads with eggs). In 2020, from 659 searched flowerheads, 53 eggs were recorded on 45 flowerheads (7% of flowerheads with eggs). Overall, accepted flowerheads had a mean of 1.93 eggs across all years. 2006 had higher eggs per flowerhead (range: 1–16, mean: 5.96), than 2007, 2008 (ranges: 1–6, means: 1.36 and 1.31 respectively), and 2020 (range: 1–3, mean: 1.18). In 2007, 2008 and 2020, the majority of accepted flowerheads had only a single egg present (75.4%, 78.1% and 88.9%, respectively), whereas in 2006 the majority of accepted flowerheads had more than one egg (2.5% had one egg) (Fig A5).

### Egg presence

**Fig. 3:**
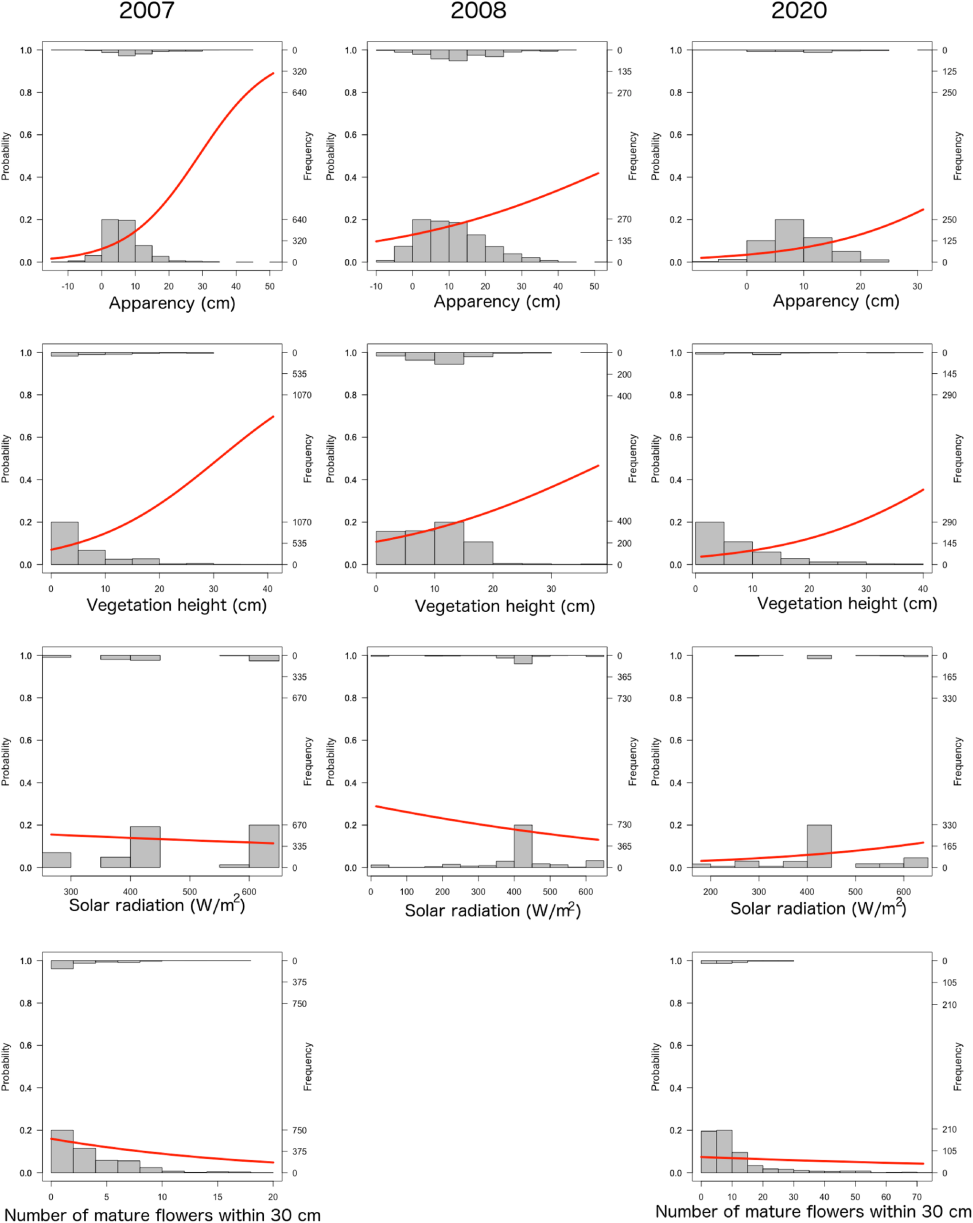
The effects of flower apparency, vegetation height, and solar radiation (recorded in all study years), and number of mature flowers within 30 cm (recorded in 2007 and 2020 only), on the probability of flowerheads being selected for oviposition by small blues (Smart et al. 2004). Columns show data collected in individual years. Data from 2006 were excluded because flowerheads without eggs were not recorded. Histogram bars show the frequency distribution of flowers with each characteristic where eggs were present (top axis) or absent (bottom axis). Red lines show predicted probabilities from best fitting models, as assessed by AIC, with other significant effects held at their means. Note: axis scales are not standardised across plots. Years are shown separately to facilitate visualisation of data, but data from all years were fitted in regression models

Small blues laid on flowerheads that were more apparent (χ² = 77.8, d.f. = 1, p < 0.001) and surrounded by taller vegetation (χ² = 106.1, d.f. = 1, p < 0.001) (Table A3, Fig. 3). The importance of apparency differed between years, with small blues showing a stronger preference for more apparent flowerheads in 2007 and 2020 than in 2008 (2007–2008: χ² = 25.60, d.f. = 1, p < 0.001; 2008–2020: χ² = 4.68, d.f. = 1, p = 0.030), but there was no difference between 2007 and 2020 (χ² = 0.064, d.f. = 1, p = 0.800). Locations with higher solar radiation were less likely to have eggs in 2007 and 2008, but more likely in 2020 (χ² = 7.3, d.f = 2, p = 0.026).

From the 2007 and 2020 data only, small blues laid on flowerheads that were more apparent (χ² = 91.20, d.f. = 1, p < 0.001), surrounded by taller vegetation (χ² = 109.92, d.f. = 1, p < 0.001), had fewer mature flowerheads nearby (χ² = 6.06, d.f. = 1, p = 0.014), and flowerheads were less likely to have eggs present in 2020 than 2007 (χ² = 19.45, d.f. = 1, p < 0.001) (Table A4, Fig. 3). Though an interaction between the number of mature flowerheads nearby and year was retained in the optimal model, it was non-significant (χ² = 0.05, d.f. = 1, p = 0.070). No difference was detected in strength of preference for apparency between years.

### Egg abundance

**Fig. 4:**
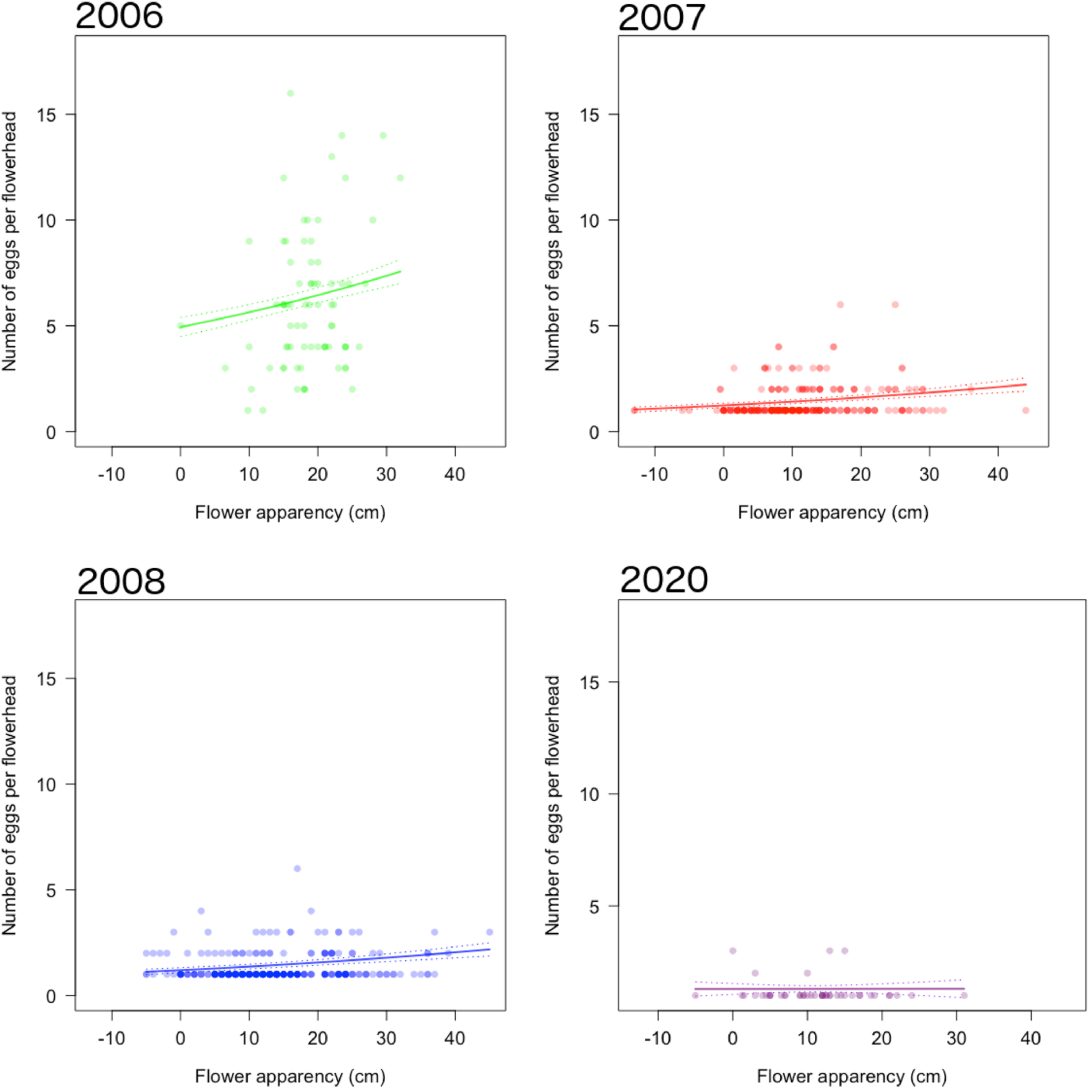
The effect of flower apparency on the number of small blue eggs per flowerhead. Points represent individual flowerheads in 2006 (green), 2007 (red), 2008 (blue), and 2020 (purple), and are semi-transparent so that overlapping points appear darker. Solid lines represent predicted values from the best fitting model, as assessed by AIC, with other significant effects held at their means. Dashed lines show standard errors. Though years are shown separately for clarity, data were fitted together in regression models. (Color figure online)

**Fig. 5:**
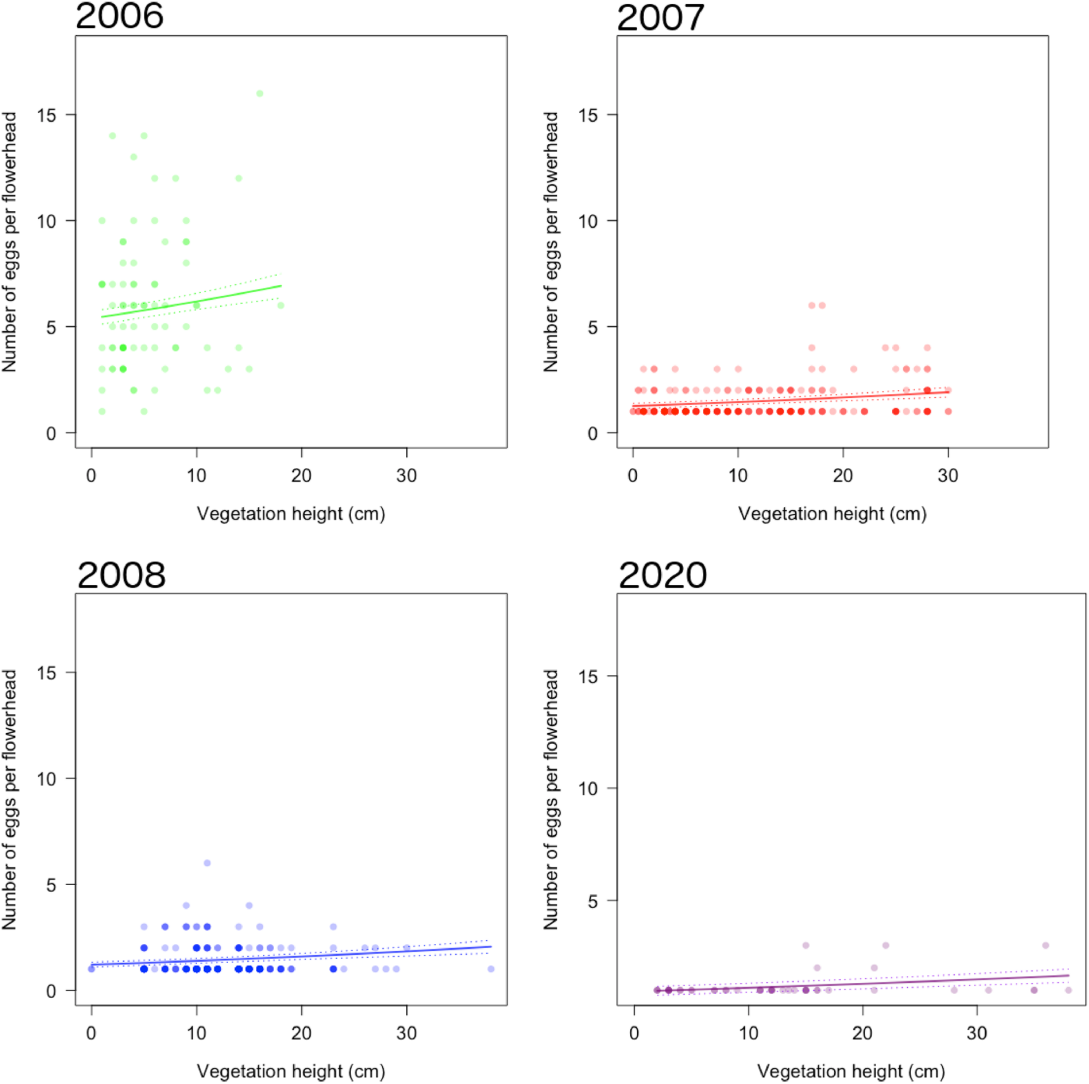
The effect of vegetation height on the number of small blue eggs per flowerhead. Points represent individual flowerheads in 2006 (green), 2007 (red), 2008 (blue), and 2020 (purple), and are semi-transparent so that overlapping points appear darker. Solid lines represent predicted values from the best fitting model, as assessed by AIC, with other significant effects held at their means. Dashed lines show standard errors. Though years are shown separately for clarity, data were fitted together in regression models. (Color figure online)

Eggs were more abundant on more apparent flowerheads (χ^2^ = 22.27, d.f. = 1, p < 0.001; Fig. 4), surrounded by taller vegetation (χ^2^ = 17.19, d.f. = 1, p < 0.001; Fig. 5). In 2006, the abundance of eggs on chosen flowerheads was higher than in subsequent years (χ^2^ = 979.60, d.f. = 3, p < 0.001; mean ± standard error: 2006 = 5.96 ± 0.36; 2007 = 1.36 ± 0.05; 2008 = 1.31 ± 0.04; 2020 = 1.18 ± 0.53 eggs per flowerhead) (Table A5). In 2006, the mean predicted egg abundance ranged from 3.5 eggs on the least apparent flowerheads (0 cm), to 9.2 eggs on the most apparent flowerheads (32 cm), compared to smaller ranges in 2007 [1.0 eggs (− 13 cm) to 2.1 eggs (44 cm)], 2008 [1.3 eggs (− 5 cm) to 1.8 eggs (45 cm)], and 2020 [1.2 eggs (− 5 cm) to 1.3 eggs (31 cm)].

In 2007 and 2020, the inclusion of solar radiation, Mature, and Buds did not improve the model (Table A6).

## Discussion

We found that adult emergence of small blues at Totternhoe has been getting earlier from 1993 to 2019. Across years, earlier emergence was associated with higher maximum temperatures in February. Annual total abundance was not predicted by monthly maximum temperatures, year, or abundance in the previous year. Egg presence was more likely, and abundance was higher, on more apparent flowerheads, surrounded by taller vegetation. Egg presence was also more likely on flowerheads surrounded by fewer other mature flowerheads. Mean solar radiation had different effects on egg presence each year, with higher solar radiation reducing the probability of egg presence in 2007 and 2008, but increasing the probability in 2020. 2006 had a higher total abundance of small blues than 2007 or 2008, and the number of eggs per flowerhead was also higher in 2006 than other years, suggesting a lack of flowerheads for ovipositing females that year. The total abundance patterns and emergence date trends of small blues recorded in Totternhoe largely reflect national trends (Fig A6) (Botham et al. 2019).

### Population trends—impacts of temperature

High February temperatures resulted in earlier emergence of small blues. This is likely to be driven by earlier breaking of diapause, earlier pupation, and more rapid development to adulthood. This mirrors results from previous studies that have identified mean temperatures between February and April as important for predicting small blue emergence date (Roy et al. 2015), and results from other butterfly species which have shown that higher temperatures reduce development time (Koda and Nakamura 2010; Fischer and Karl 2010), and influence emergence (Davies 2019).

A reduced development time can be beneficial for survival. The slow growth-high mortality hypothesis (Feeny 1976) predicts that faster development is favoured because it reduces the time spent as a larva which is vulnerable to predators and parasitoids. However, there is limited evidence that this hypothesis is supported for species that have concealed feeding or pupation sites (Atlegrim 1992; Tvardikova and Novotny 2012). As small blue larvae overwinter in concealed locations on the ground before pupating in April (Thomas and Lewington 2016), they are unlikely to be seen by natural enemies, potentially reducing predation pressure and weakening any relationship between development rate and risk of predation.

Alternatively, increased development rate may increase the potential for a mismatch between emergence of the butterfly and flowering of kidney vetch (Thackeray et al. 2010). Small blue larvae are cannibalistic, so a reduction in the number of flowers at a suitable development stage for egg-laying would increase competition for this resource, resulting in increased larval mortality. To exacerbate this issue, it is not only the small blue larvae that use kidney vetch flowers. Small blue adults are also highly dependent on kidney vetch as nectaring source (Hardy et al. 2007). Although some interacting species (such as larvae and their host plants) use the same cues and respond similarly to changing temperatures, not all species pairs respond in the same way (Cook et al. 2012; Thompson and Gilbert 2014). Indeed, previous studies have found substantial variation in sensitivity and responses to temperature between interacting butterflies and plants (Gordo and Sanz 2005). Although February temperature has been found to be the best predictor of flowering time across a range of British flowering species (Fitter et al. 1995), further study is needed to quantify this effect on kidney vetch specifically.

Total adult abundance of small blues was not affected by temperature prior to emergence. This lack of effect is surprising, as we predicted temperature would have an effect on survival of larvae and pupae, as well as detectability of adults (Roy et al. 2001; Turlure et al. 2011). It is likely that this lack of effect is due to high inter-annual variation in small blue numbers at our site, as is observed at both the national scale (Botham et al. 2019), which may mask any relationships (Fig A3, A6).

### Population trends—change over time

We found that small blue emergence date at Totternhoe has got earlier over time. This pattern could be driven by climatic changes not captured by mean monthly maximum temperature, such as rainfall or extreme weather events. Rainfall was not included in our models due to inaccessibility of local data and to avoid overfitting, but has been shown to have both positive and negative effects on butterfly emergence (Stefanescu et al. 2003). Extreme weather events have also been shown to impact butterfly emergence (Patterson et al. 2020), although this is unlikely to have driven the linear increase in emergence over time that we observed. In addition, unseasonally high temperatures would have been captured by mean monthly maximum temperature, as this correlates strongly with the highest recorded temperature per month (cor = 0.944, p < 0.001).

In contrast to emergence date, total numbers of small blues did not change over time in this site, nor was it related abundance in the previous year. This finding reflects results from previous long-term studies, where despite changes in distribution being detected, no significant changes in abundance over time have yet been detected for the small blue (Fox et al. 2015; Macgregor et al. 2019), and suggests that the population at our site is stable in the long-term.

### Oviposition behaviour

We found that oviposition was more likely on more apparent flowers, with taller surrounding vegetation, and fewer nearby mature flowerheads. In addition, solar radiation affected the likelihood of eggs being present, but this effect changed over time, with higher radiation being negatively associated with egg presence in 2007 and 2008, but positively associated in 2020. We also found that egg abundance was higher on more apparent flowers with taller surrounding vegetation, implying that relatively consistent cues are used for oviposition by females, and that multiple females sometimes select the same flower for oviposition, despite the risk of cannibalism. However this is rare in most years, with the majority of flowers in 2007, 2008, and 2020 having a single egg present, but with 2006 being an exception to this.

The relationship between egg abundance and flower apparency and the number of mature flowers are likely to either be the result of detection bias by the butterflies, a pattern which has been observed previously (Morton 1985), or because these features are indicators of host plant quality. An apparent flowerhead is more likely to be detected by egg-laying females, while a flowerhead with fewer nearby flowerheads is more likely to be selected as there is limited choice of alternative sites in the immediate vicinity. Alternatively, females may be actively choosing more apparent flowerheads because of beneficial characteristics. Although small blues have a short adult lifespan of approximately 15 days (Bubova et al. 2016), which reduces their ability to be selective in oviposition, they also have relatively low egg production (estimated 40 eggs per female) (Leon-Cortes et al. 2003), which may increase selectivity of egg-laying sites (Doak et al. 2006). Females may choose larger flowerheads growing in taller vegetation because such flowerheads have more or larger seeds, or be of higher quality, and therefore provide larvae with more resources. Another possibility is that tall vegetation provides a more favourable microclimate, including lower (Valdés and Ehrlén 2018) and less variable temperature (Green et al. 1984; Song et al. 2013), which could reduce energy use and increase larval survival. Finally, the higher structural complexity in tall vegetation could provide defence against predation (Atkinson et al. 2004) and parasitism (Obermaier et al. 2008), potentially increasing larval survival. There has been limited work on the predators and parasites of small blues, however low levels of egg predation by ants have been recorded, which was most prevalent on short-stemmed kidney vetch, and larvae have been recorded being attacked by the parasitoids *Diadegma aculeats* and *Agathis* species (Hymenoptera) (Morton 1985), suggesting that factors reducing predator and parasitoid attack could be benefit larval survival. We are not aware of any work that has investigated whether taller kidney vetch flowerheads have larger or more seeds and, therefore, more resources for the larvae, or whether taller vegetation confers any of the other possible advantages to small blue larvae posited here. More research is therefore required.

We found that the mean solar radiation a flowerhead received affected egg-laying behaviour, although the direction of this preference differed between years. Mean solar radiation is highest on south-facing slopes, and lowest on north-facing slopes (Rorison et al. 1986), and high solar radiation results in hotter and drier conditions (Bennie et al. 2006). In 2007 and 2008, egg-laying females selected flowerheads receiving lower solar radiation, whereas in 2020 they selected flowerheads receiving higher solar radiation. This may be the result of inter-annual variation in temperature. If this were the case, egg distribution should favour host plant patches with higher solar radiation in cooler years, and vice versa. However, 2007 and 2008 had lower temperatures during the egg-laying period (late May–early July: mean temperature of 17.4 °C and 17.2 °C, respectively) than 2020 (19.1 °C), making this explanation unlikely. Alternatively, this result could be due to other factors that vary between years, such as rainfall. High solar radiation increases plant respiration (Fitter et al. 1998), evapotranspiration, and water loss (Liu et al. 2009) and previous studies of Lycaenids have found oviposition preferences for host plants with higher water content (Wagner and Kurina, 1997; Pickens and Root 2008). The patches receiving higher solar radiation are likely to dry out faster, particularly in porous calcareous soil, so it is possible that flowerheads with lower solar radiation may be selected preferentially in drier years. Finally, it is important to consider that not all areas were searched equally across all years, so it is possible this finding is the result of an observation bias, where locations with lower solar radiation were searched more often in 2007 and 2008 by chance.

Whether detection bias or active selection are the key drivers, the net result is that only a small subset of tall kidney vetch flowerheads with tall surrounding vegetation are used for egg-laying. These trends were relatively consistent across our study years, despite substantial variation in temperature and adult abundance between years. However, there was variation in the strength of preference for highly apparent flowers, and a change in direction of preference for solar radiation. This implies that other inter-annual variation, such as the abundance of suitable flowerheads or climatic conditions (Berger et al. 2008), may affect oviposition preferences. Nonetheless, the consistent direction of preference for flowerhead characteristics suggests that management for these characteristics will be effective across sites and years.

Although trends in the choice of flowerheads were similar, we detected large differences in the number of eggs laid per accepted flowerhead between years, with egg abundance per flowerhead in 2006 being much higher than in other years. This difference could be related to several factors: a higher abundance of small blue adults, a lower abundance of kidney vetch, or a mismatch between kidney vetch flowering and small blue emergence, leading to high small blue numbers before kidney vetch flowerheads were developed. There is support for all three of these explanations in 2006, with a the year having a high abundance of small blues, an dry and cold winter which could have reduced kidney vetch seedling survival and led to a mismatch between flowering and butterfly emergence, and low summer rain (Met Office 2006), which reduces kidney vetch growth and seed production (Davison et al. 2010). A similar pattern was recorded in Belgium after a severe summer heatwave and drought in 2003, when kidney vetch abundance declined, resulting in high small blue egg abundance per flowerhead in 2004 (Piessens et al. 2009). This particular event is also reflected in Totternhoe, with a decline in small blue abundance from 2003 to 2004 (Fig A3). As small blue larvae are cannibalistic (Asher et al. 2001), this is likely to have led to substantial mortality, and may explain the population crash observed the following year (Fig A3). Morton (1985) reported a similar pattern in 1983 in a site in West Sussex, UK, where some flowers received over 50 eggs, and approximately 1000 larvae died as a result of cannibalism, resulting in a population crash in 1984.

### Conclusions and management implications

This study has highlighted the influence of spring temperature variation on small blue emergence date, as well as the importance of kidney vetch flowerhead apparency and vegetation height in predicting small blue oviposition. This study has also highlighted a change in direction of preference for solar radiation, which may reflect differences in rainfall during egg-laying periods between years. Our study demonstrates the value of collecting long-term data from well-studied sites, allowing fine-scale changes to be assessed at the reserve scale. Spring temperatures are predicted to increase under climate change (IPCC 2014), which will continue to impact small blue emergence, and potentially pose a threat to the persistence of the butterfly on many sites.

Based on our results, management should aim to reduce the number of eggs per flowerhead and increase larval survival. This could be done by producing a high abundance of suitable flowerheads, by protecting kidney vetch patches from cutting and grazing during spring to promote growth of taller flowers amongst taller vegetation, alongside disturbing other areas to promote new kidney vetch propagation for subsequent years. Our results indicate that multiple eggs on single flowerheads is primarily an issue in high-abundance years. Despite most flowers not being used in most years, the density of eggs at Totternhoe Quarry in 2006 highlights the importance of managing for a suitable number of oviposition sites in peak years. This is likely to reduce cannibalism in high abundance years, reduce the chance of a subsequent population crash, increase the long-term persistence of a population, and increase the probability of individuals colonising new areas.

## Supplementary Information

Below is the link to the electronic supplementary material.

Supplementary material 1 (DOCX 1124.5 kb)
