# Supplementary material for: Oviposition behaviour and emergence through time of the small blue butterfly (Cupido minimus) in a nature reserve in Bedfordshire, UK
Source: J Insect Conserv. 2021 Dec 6;26(1):43–58. doi: 10.1007/s10841-021-00360-5 (PMC8647801; doi:10.1007/s10841-021-00360-5)
Supplement: Supplementary file 1 — Supplementary material 1 (DOCX 1124.5 kb) [file 10841_2021_360_MOESM1_ESM.docx]

**Oviposition behaviour and emergence through time of the small blue butterfly (*Cupido minimus*) in a nature reserve in Bedfordshire, UK**

**Supplementary Materials**

Esme Ashe-Jepson^1^, Andrew J. Bladon^1^, Greg Herbert^2^, Gwen E. Hitchcock^3^, Richard Knock^3^, Colin B. Lucas^4^, Sarah H. Luke^1^ & Edgar C. Turner^1^

**Affiliations**

1. Department of Zoology, University of Cambridge, Downing Street, Cambridge, CB2 3EJ, UK. [ea483@cam.ac.uk](mailto:ea483@cam.ac.uk), [ajb273@cam.ac.uk](mailto:ajb273@cam.ac.uk), [shl47@cam.ac.uk](mailto:shl47@cam.ac.uk), [ect23@cam.ac.uk](mailto:ect23@cam.ac.uk)
2. Bedfordshire and Northamptonshire branch of Butterfly Conservation. Registered office: Manor Yard, East Lulworth, Wareham, Dorset, BH20 5QP, UK. Gregherbert1@gmail.com
3. The Wildlife Trust for Bedfordshire, Cambridgeshire and Northamptonshire, The Manor House, Broad Street, Cambourne, Cambridge, CB23 6DH, UK. [gwen.hitchcock@wildlifebcn.org](mailto:gwen.hitchcock@wildlifebcn.org); [richard.knock@wildlifebcn.org](mailto:richard.knock@wildlifebcn.org)
4. 49 Mill Road, Beccles, Suffolk, NR34 9UT, UK. [colinbh@hotmail.co.uk](mailto:colinbh@hotmail.co.uk)

**
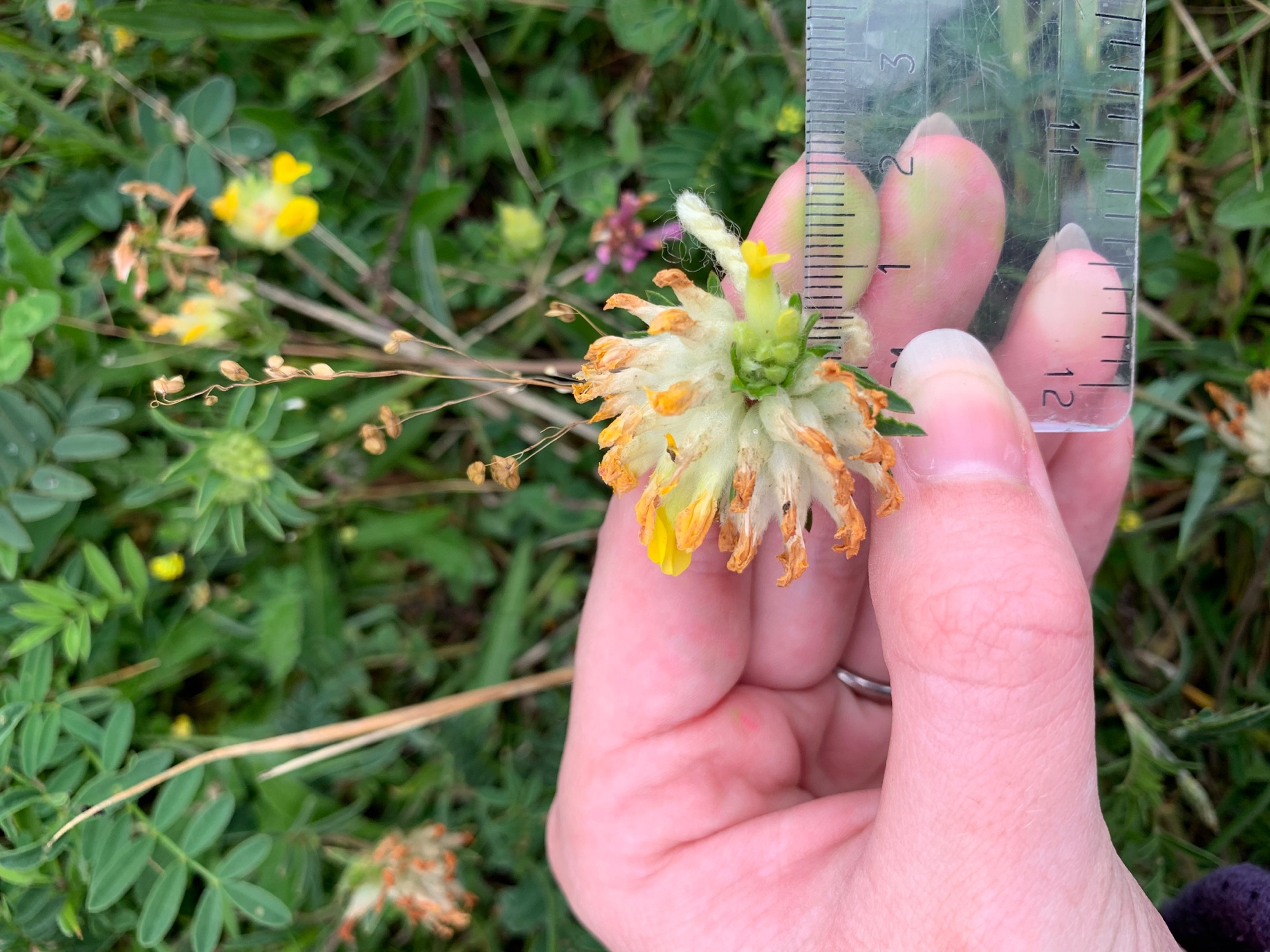
**

Figure A1: A photograph of a kidney vetch flowerhead being searching for small blue eggs. An egg is highlighted with the black arrow.


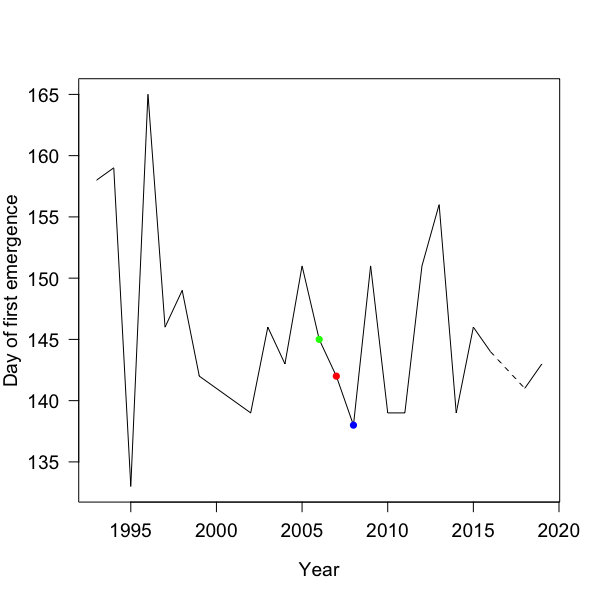
Figure A2: Day of first emergence of small blue adults at Totternhoe Quarry from 1993-2019. Coloured points represent years when detailed data on oviposition preferences were collected (green = 2006, red = 2007, blue = 2008). 2020 not marked as emergence data were only available up until 2019. Data were not available for 2017 (dashed line). Data from the UKBMS (Brereton et al. 2019), made available through the Bedfordshire and Luton Biodiversity Recording and Monitoring Centre (2020).


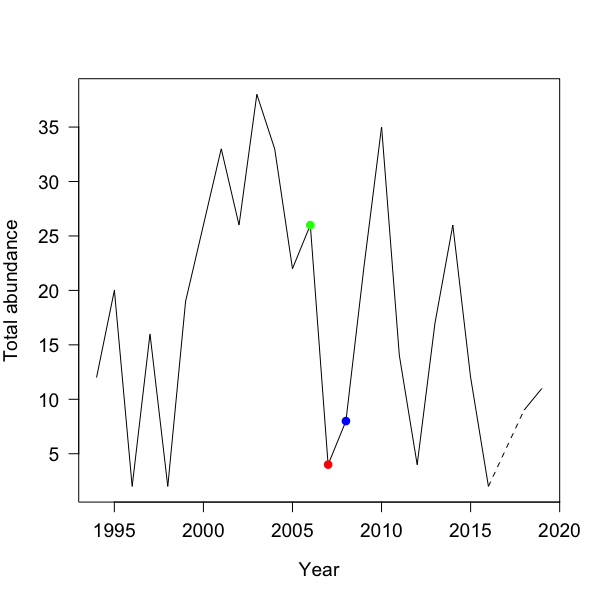
Figure A3: Adult small blue total abundance across years at Totternhoe Quarry, from 1993-2019. Coloured points represent years when detailed data on oviposition preferences were collected (green = 2006, red = 2007, blue = 2008). 2020 not marked as abundance data were only available up until 2019. Data were not available for 2017 (dashed line). Data from the UKBMS (Brereton et al. 2019) made available through the Bedfordshire and Luton Biodiversity Recording and Monitoring Centre (2020).


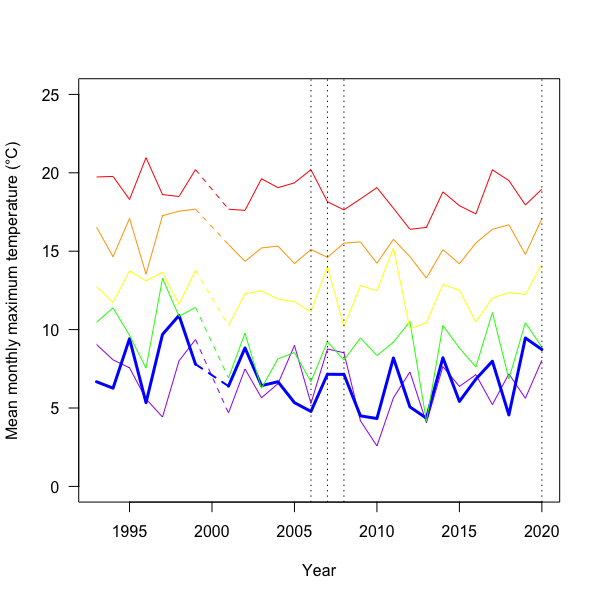


Figure A4: Mean monthly maximum temperature at Woburn weather station (13.9 km from Totternhoe Quarry) across years, 1993-2020. Lines are months: January = purple, February = blue, March = green, April = yellow, May = orange, June = red. February line is bolded as temperature in this month was found to be a significant predictor of small blue first emergence. Vertical dotted lines highlight years in which oviposition data were collected (2006, 2007, 2008, 2020). Data were not available in 2000 from this weather station (dashed lines). Data from the Met Office (2020).


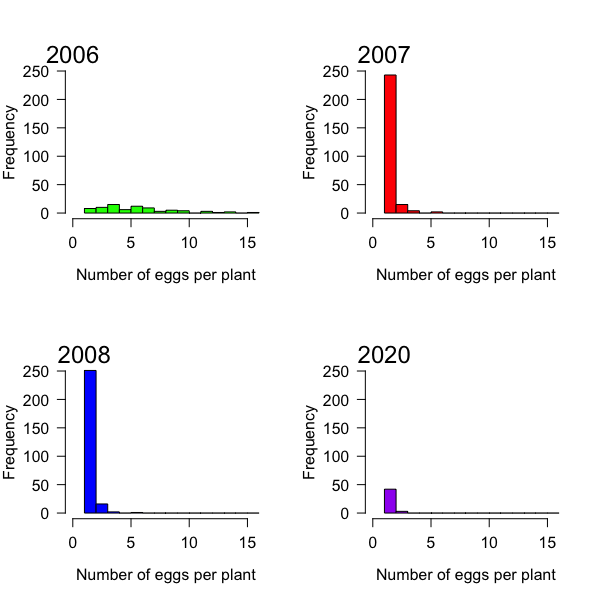


Figure A5: Frequency distributions of the number of small blue eggs recorded per kidney vetch flowerhead, split by study year.


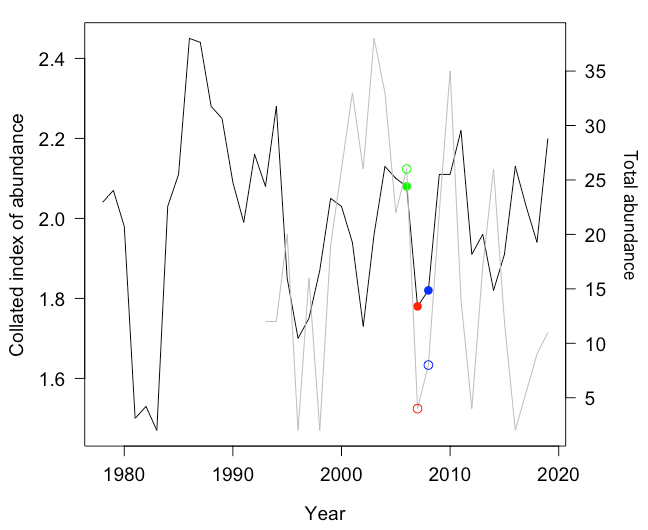


Figure A6: Collated index of abundance of small blue adults over time (1978-2019), at the national (UK) scale. Total yearly abundance in Totternhoe quarry on a separate axis in grey. Coloured points represent years when detailed data on oviposition preferences were collected (green = 2006, red = 2007, blue = 2008). Collated indices are produced by the UK Butterfly Monitoring Scheme (Botham et al. 2019). Total abundance data from the UKBMS (Brereton et al. 2019) made available through the Bedfordshire and Luton Biodiversity Recording and Monitoring Centre (2020).

Table A1: Model selection table with multiple linear regressions for emergence date, with main effects (average maximum temperature in January-June, and year (1993-2019)) listed. Variables included in each model are denoted with a ‘+’. Models are ordered by AICc, with the optimal model in bold. Only the top 50 models are shown.

| January | February | March | April | May | June | Year | d.f. | AICc | ΔAICc | Number of factors |
| --- | --- | --- | --- | --- | --- | --- | --- | --- | --- | --- |
| **-** | **+** | **-** | **-** | **-** | **-** | **+** | **4** | **164.3** | **0.0** | **2** |
| - | + | - | - | + | - | + | 5 | 165.0 | 0.7 | 3 |
| - | - | - | - | + | - | + | 4 | 165.4 | 1.1 | 2 |
| - | + | - | + | - | - | + | 5 | 166.0 | 1.7 | 3 |
| - | - | - | + | + | - | + | 5 | 166.8 | 2.5 | 3 |
| - | + | + | - | - | - | + | 5 | 167.3 | 3.0 | 3 |
| - | + | - | + | + | - | + | 6 | 167.4 | 3.1 | 4 |
| - | + | - | - | - | + | + | 5 | 167.5 | 3.2 | 3 |
| + | + | - | - | - | - | + | 5 | 167.6 | 3.3 | 3 |
| - | + | + | - | + | - | + | 6 | 167.8 | 3.5 | 4 |
| - | - | - | - | + | + | + | 5 | 168.0 | 3.7 | 3 |
| + | + | - | - | + | - | + | 6 | 168.6 | 4.3 | 4 |
| - | + | - | - | + | + | + | 6 | 168.6 | 4.3 | 4 |
| + | - | - | - | + | - | + | 5 | 168.6 | 4.3 | 3 |
| - | + | - | - | - | - | - | 3 | 168.6 | 4.3 | 1 |
| - | - | + | - | + | - | + | 5 | 168.6 | 4.3 | 3 |
| - | - | - | - | + | + | - | 4 | 169.0 | 4.7 | 2 |
| - | - | - | + | + | + | + | 6 | 169.0 | 4.7 | 4 |
| - | + | + | + | - | - | + | 6 | 169.0 | 4.7 | 4 |
| - | - | - | + | - | - | + | 4 | 169.2 | 4.9 | 2 |
| - | - | - | - | + | - | - | 3 | 169.5 | 5.2 | 1 |
| + | + | - | + | - | - | + | 6 | 169.5 | 5.2 | 4 |
| - | - | - | - | - | - | + | 3 | 169.5 | 5.2 | 1 |
| - | + | - | + | - | + | + | 6 | 169.6 | 5.3 | 4 |
| - | - | - | - | - | - | - | 2 | 169.7 | 5.4 | 0 |
| - | + | - | - | - | + | - | 4 | 170.0 | 5.7 | 2 |
| - | - | - | + | - | + | - | 4 | 170.1 | 5.8 | 2 |
| + | - | - | + | + | - | + | 6 | 170.2 | 5.9 | 4 |
| - | + | + | + | + | - | + | 7 | 170.2 | 5.9 | 5 |
| - | + | + | - | - | - | - | 4 | 170.2 | 5.9 | 2 |
| - | - | - | + | + | + | - | 5 | 170.3 | 6.0 | 3 |
| - | - | + | + | + | - | + | 6 | 170.3 | 6.0 | 4 |
| - | - | - | - | - | + | - | 3 | 170.4 | 6.1 | 1 |
| - | + | + | - | - | + | + | 6 | 170.8 | 6.5 | 4 |
| - | + | - | - | + | - | - | 4 | 170.9 | 6.6 | 2 |
| + | + | + | - | - | - | + | 6 | 170.9 | 6.6 | 4 |
| - | - | - | + | - | - | - | 3 | 171.0 | 6.7 | 1 |
| + | + | - | - | - | + | + | 6 | 171.0 | 6.7 | 4 |
| - | - | - | + | - | + | + | 5 | 171.1 | 6.8 | 3 |
| - | + | - | + | - | - | - | 4 | 171.2 | 6.9 | 2 |
| - | - | + | - | - | - | + | 4 | 171.2 | 6.9 | 2 |
| - | + | - | + | + | + | + | 7 | 171.2 | 6.9 | 5 |
| + | + | - | - | - | - | - | 4 | 171.3 | 7.0 | 2 |
| + | - | - | + | - | - | + | 5 | 171.4 | 7.1 | 3 |
| + | + | - | + | + | - | + | 7 | 171.4 | 7.1 | 5 |
| - | + | - | - | + | + | - | 5 | 171.6 | 7.3 | 3 |
| + | - | - | - | + | + | + | 6 | 171.6 | 7.3 | 4 |
| - | - | + | - | + | + | + | 6 | 171.6 | 7.3 | 4 |
| - | - | + | - | + | + | - | 5 | 171.6 | 7.3 | 3 |
| - | + | - | + | - | + | - | 5 | 171.7 | 7.4 | 3 |

Table A2: Model selection table with negative binomial Generalised Linear Models for total abundance, with main effects (average maximum temperature in January-June, year (1993-2019), and total abundance of the previous year) listed. Variables included in each model are denoted with a ‘+’. Models are ordered by AICc, with the optimal model in bold. Only the top 50 models are shown.

| January | February | March | April | May | June | Year | Previous year total abundance | d.f. | AICc | ΔAICc | Number of factors |
| --- | --- | --- | --- | --- | --- | --- | --- | --- | --- | --- | --- |
| + | - | - | - | - | - | - | - | 3 | 185.4 | 0.0 | 1 |
| **-** | **-** | **-** | **-** | **-** | **-** | **-** | **-** | **2** | **185.4** | **0.0** | **0** |
| + | - | - | - | - | - | - | + | 4 | 185.6 | 0.2 | 2 |
| - | - | - | - | - | - | - | + | 3 | 185.7 | 0.3 | 1 |
| + | - | - | - | - | + | - | - | 4 | 186.4 | 1.0 | 2 |
| + | - | - | - | - | + | - | + | 5 | 186.9 | 1.5 | 3 |
| - | - | + | - | - | - | - | - | 3 | 187.0 | 1.6 | 1 |
| + | - | - | - | - | - | + | - | 4 | 187.1 | 1.7 | 2 |
| - | - | - | - | - | + | - | - | 3 | 187.1 | 1.7 | 1 |
| - | + | - | - | - | - | - | - | 3 | 187.5 | 2.1 | 1 |
| + | - | - | - | - | - | + | + | 5 | 187.6 | 2.2 | 3 |
| - | - | - | - | - | - | + | - | 3 | 187.6 | 2.2 | 1 |
| + | - | - | - | + | - | - | + | 5 | 187.6 | 2.2 | 3 |
| - | - | - | - | - | + | - | + | 4 | 187.9 | 2.5 | 2 |
| - | - | - | - | + | - | - | - | 3 | 187.9 | 2.5 | 1 |
| - | - | - | + | - | - | - | - | 3 | 188.0 | 2.6 | 1 |
| + | - | + | - | - | - | - | - | 4 | 188.1 | 2.7 | 2 |
| + | - | - | + | - | - | - | - | 4 | 188.1 | 2.7 | 2 |
| - | + | - | - | - | - | - | + | 4 | 188.2 | 2.8 | 2 |
| - | - | + | - | - | - | - | + | 4 | 188.2 | 2.8 | 2 |
| - | - | - | - | - | - | + | + | 4 | 188.2 | 2.8 | 2 |
| + | - | - | - | + | - | - | - | 4 | 188.3 | 2.9 | 2 |
| + | + | - | - | - | - | - | - | 4 | 188.3 | 2.9 | 2 |
| - | - | - | - | + | - | - | + | 4 | 188.5 | 3.1 | 2 |
| + | - | - | + | - | - | - | + | 5 | 188.5 | 3.1 | 3 |
| - | - | - | + | - | - | - | + | 4 | 188.6 | 3.2 | 2 |
| - | - | + | - | - | + | - | - | 4 | 188.8 | 3.4 | 2 |
| + | + | - | - | - | - | - | + | 5 | 188.8 | 3.4 | 3 |
| + | - | + | - | - | - | - | + | 5 | 188.8 | 3.4 | 3 |
| + | - | - | - | - | + | + | - | 5 | 189.0 | 3.6 | 3 |
| - | - | + | - | - | - | + | - | 4 | 189.0 | 3.6 | 2 |
| - | - | + | + | - | - | - | - | 4 | 189.1 | 3.8 | 2 |
| + | - | + | - | - | + | - | - | 5 | 189.4 | 4.0 | 3 |
| - | + | - | - | - | - | + | - | 4 | 189.5 | 4.1 | 2 |
| + | + | - | - | - | + | - | - | 5 | 189.6 | 4.2 | 3 |
| + | - | - | - | + | + | - | - | 5 | 189.6 | 4.2 | 3 |
| - | - | - | - | + | + | - | - | 4 | 189.6 | 4.2 | 2 |
| + | - | - | + | - | + | - | - | 5 | 189.6 | 4.2 | 3 |
| - | + | - | - | - | + | - | - | 4 | 189.6 | 4.3 | 2 |
| + | - | + | - | - | - | + | - | 5 | 189.8 | 4.4 | 3 |
| - | - | - | - | - | + | + | - | 4 | 189.8 | 4.4 | 2 |
| + | - | - | - | - | + | + | + | 6 | 189.9 | 4.5 | 4 |
| - | - | + | - | + | - | - | - | 4 | 189.9 | 4.5 | 2 |
| - | + | + | - | - | - | - | - | 4 | 189.9 | 4.5 | 2 |
| - | - | - | + | - | + | - | - | 4 | 190.0 | 4.6 | 2 |
| - | - | - | - | + | - | + | - | 4 | 190.0 | 4.6 | 2 |
| + | - | - | - | + | + | - | + | 6 | 190.0 | 4.7 | 4 |
| - | + | - | + | - | - | - | - | 4 | 190.1 | 4.7 | 2 |
| + | - | - | + | - | - | + | - | 5 | 190.2 | 4.8 | 3 |
| + | + | - | - | - | - | + | - | 5 | 190.2 | 4.8 | 3 |

Table A3: Model selection table with binomial Generalised Linear Models of egg presence in all years (2007, 2008, 2020), with main effects and interaction effects listed. Variables included in each model are denoted with a ‘+’. Models ordered by AIC, with the optimal model in bold. All possible models are shown.

| Main effects | | | | Interaction effects | | |  |  |  |  |
| --- | --- | --- | --- | --- | --- | --- | --- | --- | --- | --- |
| Apparency | Year | Solar radiation | Vegetation height | Year: Apparency | Year: Solar radiation | Year: Vegetation height | df | AIC | ΔAIC | Number of parameters |
| + | + | + | + | + | + | + | 12 | 3085.5 | 0 | 7 |
| **+** | **+** | **+** | **+** | **+** | **+** | **-** | **10** | **3086.7** | **1.22** | **6** |
| + | + | - | + | + | - | + | 9 | 3088.2 | 2.66 | 5 |
| + | + | - | + | + | - | - | 7 | 3089.2 | 3.71 | 4 |
| + | + | + | + | + | - | + | 10 | 3089.3 | 3.76 | 6 |
| + | + | + | + | + | - | - | 8 | 3090 | 4.48 | 5 |
| + | + | + | + | - | + | + | 10 | 3108.4 | 22.94 | 6 |
| + | + | - | + | - | - | + | 7 | 3109.6 | 24.1 | 4 |
| + | + | + | + | - | + | - | 8 | 3110.7 | 25.18 | 5 |
| + | + | - | + | - | - | - | 5 | 3111.2 | 25.71 | 3 |
| + | + | + | + | - | - | + | 8 | 3111.5 | 25.95 | 5 |
| + | + | + | + | - | - | - | 6 | 3112.9 | 27.38 | 4 |
| + | - | - | + | - | - | - | 3 | 3141.9 | 56.43 | 2 |
| + | - | + | + | - | - | - | 4 | 3143.7 | 58.24 | 3 |
| - | + | + | + | - | + | + | 9 | 3184.7 | 99.15 | 5 |
| - | + | + | + | - | + | - | 7 | 3186.5 | 100.98 | 4 |
| + | + | + | - | + | + | - | 9 | 3190.8 | 105.29 | 5 |
| - | + | - | + | - | - | + | 6 | 3191 | 105.48 | 3 |
| - | + | - | + | - | - | - | 4 | 3191.5 | 105.98 | 2 |
| - | + | + | + | - | - | + | 7 | 3192.5 | 106.96 | 4 |
| - | + | + | + | - | - | - | 5 | 3192.6 | 107.06 | 3 |
| + | + | + | - | + | - | - | 7 | 3196.2 | 110.66 | 4 |
| + | + | - | - | + | - | - | 6 | 3202 | 116.46 | 3 |
| + | + | + | - | - | + | - | 7 | 3217.8 | 132.26 | 4 |
| + | + | + | - | - | - | - | 5 | 3222.4 | 136.85 | 3 |
| + | + | - | - | - | - | - | 4 | 3225.6 | 140.09 | 2 |
| - | - | - | + | - | - | - | 2 | 3229.6 | 144.12 | 1 |
| - | - | + | + | - | - | - | 3 | 3229.7 | 144.19 | 2 |
| + | - | + | - | - | - | - | 3 | 3251 | 165.49 | 2 |
| + | - | - | - | - | - | - | 2 | 3254.4 | 168.89 | 1 |
| - | + | + | - | - | + | - | 6 | 3303 | 217.49 | 3 |
| - | + | + | - | - | - | - | 4 | 3309.2 | 223.72 | 2 |
| - | + | - | - | - | - | - | 3 | 3314.9 | 229.39 | 1 |
| - | - | + | - | - | - | - | 2 | 3352.1 | 266.63 | 1 |
| - | - | - | - | - | - | - | 1 | 3362.5 | 277.02 | 0 |

Table A4: Model selection table with binomial Generalised Linear Models of egg presence for 2007 and 2020, with main effects and interaction effects listed. Included variables are denoted with a ‘+’. Ordered by AIC, with the optimal model in bold. Only the top 50 models are shown.

| Main effects | | | | | | Interaction effects | | | | |  |  |  |  |
| --- | --- | --- | --- | --- | --- | --- | --- | --- | --- | --- | --- | --- | --- | --- |
| Apparency | Year | Buds | Mean solar radiation | Mature | Vegetation height | Year: Apparency | Year: Buds | Year: Solar radiation | Year:Mature | Year: Vegetation height | df | AIC | ΔAIC | Number of parameters |
| + | + | + | - | + | + | - | - | - | + | - | 7 | 1687.9 | 0 | 6 |
| **+** | **+** | **-** | **-** | **+** | **+** | **-** | **-** | **-** | **+** | **-** | **6** | **1688** | **0.12** | **5** |
| + | + | + | + | + | + | - | - | + | + | - | 9 | 1688.7 | 0.89 | 8 |
| + | + | + | - | + | + | - | - | - | - | - | 6 | 1688.8 | 0.92 | 5 |
| + | + | + | - | + | + | - | + | - | + | - | 8 | 1688.8 | 0.97 | 7 |
| + | + | - | + | + | + | - | - | + | + | - | 8 | 1688.8 | 0.98 | 7 |
| + | + | - | - | + | + | - | - | - | - | - | 5 | 1689.3 | 1.49 | 4 |
| + | + | - | + | + | + | - | - | - | + | - | 7 | 1689.4 | 1.52 | 6 |
| + | + | + | + | + | + | - | - | - | + | - | 8 | 1689.5 | 1.66 | 7 |
| + | + | + | - | + | + | + | - | - | + | - | 8 | 1689.6 | 1.74 | 7 |
| + | + | + | + | + | + | - | - | + | - | - | 8 | 1689.6 | 1.76 | 7 |
| + | + | - | - | + | + | + | - | - | + | - | 7 | 1689.7 | 1.85 | 6 |
| + | + | + | - | + | + | - | - | - | + | + | 8 | 1689.8 | 1.9 | 7 |
| + | + | - | - | + | + | - | - | - | + | + | 7 | 1689.8 | 1.95 | 6 |
| + | + | + | + | + | + | - | + | + | + | - | 10 | 1689.9 | 2.08 | 9 |
| + | + | + | - | + | + | - | + | - | - | - | 7 | 1690 | 2.19 | 6 |
| + | + | - | + | + | + | - | - | + | - | - | 7 | 1690.2 | 2.3 | 6 |
| + | + | + | + | + | + | - | - | - | - | - | 7 | 1690.4 | 2.58 | 6 |
| + | + | + | + | + | + | + | - | + | + | - | 10 | 1690.4 | 2.59 | 9 |
| + | + | + | - | + | + | + | - | - | - | - | 7 | 1690.5 | 2.62 | 6 |
| + | + | - | + | + | + | + | - | + | + | - | 9 | 1690.5 | 2.65 | 8 |
| + | + | + | + | + | + | - | + | - | + | - | 9 | 1690.5 | 2.65 | 8 |
| + | + | - | + | + | + | - | - | + | + | + | 9 | 1690.5 | 2.68 | 8 |
| + | + | + | + | + | + | - | - | + | + | + | 10 | 1690.6 | 2.71 | 9 |
| + | + | + | - | + | + | - | + | - | + | + | 9 | 1690.6 | 2.77 | 8 |
| + | + | + | - | + | + | + | + | - | + | - | 9 | 1690.7 | 2.82 | 8 |
| + | + | + | - | + | + | - | - | - | - | + | 7 | 1690.7 | 2.87 | 6 |
| + | + | - | + | + | + | - | - | - | - | - | 6 | 1690.7 | 2.87 | 5 |
| + | + | - | - | + | + | + | - | - | - | - | 6 | 1691 | 3.18 | 5 |
| + | + | - | + | + | + | + | - | - | + | - | 8 | 1691 | 3.18 | 7 |
| + | + | + | + | + | + | - | + | + | - | - | 9 | 1691.1 | 3.2 | 8 |
| + | + | - | + | + | + | - | - | - | + | + | 8 | 1691.1 | 3.21 | 7 |
| + | + | + | + | + | + | + | - | - | + | - | 9 | 1691.2 | 3.35 | 8 |
| + | + | - | - | + | + | - | - | - | - | + | 6 | 1691.2 | 3.38 | 5 |
| + | + | + | + | + | + | + | - | + | - | - | 9 | 1691.3 | 3.42 | 8 |
| + | + | + | + | + | + | - | - | - | + | + | 9 | 1691.3 | 3.47 | 8 |
| + | + | + | + | + | + | - | - | + | - | + | 9 | 1691.5 | 3.66 | 8 |
| + | + | + | - | + | + | + | - | - | + | + | 9 | 1691.5 | 3.69 | 8 |
| + | + | - | - | + | + | + | - | - | + | + | 8 | 1691.6 | 3.75 | 7 |
| + | + | + | + | + | + | - | + | + | + | + | 11 | 1691.6 | 3.79 | 10 |
| + | + | + | + | + | + | - | + | - | - | - | 8 | 1691.7 | 3.86 | 7 |
| + | + | + | + | + | + | + | + | + | + | - | 11 | 1691.8 | 3.9 | 10 |
| + | + | - | + | + | + | + | - | + | - | - | 8 | 1691.8 | 3.91 | 7 |
| + | + | + | - | + | + | + | + | - | - | - | 8 | 1691.8 | 3.99 | 7 |
| + | + | + | - | - | + | - | - | - | - | - | 5 | 1691.9 | 4.01 | 4 |
| + | + | + | - | + | + | - | + | - | - | + | 8 | 1691.9 | 4.07 | 7 |
| + | + | - | + | + | + | - | - | + | - | + | 8 | 1692 | 4.1 | 7 |
| + | + | + | + | + | + | + | - | - | - | - | 8 | 1692.1 | 4.22 | 7 |
| + | + | + | + | + | + | - | + | - | + | + | 10 | 1692.2 | 4.34 | 9 |
| + | + | - | + | + | + | + | - | + | + | + | 10 | 1692.3 | 4.45 | 9 |

Table A5: Model selection table with Conway-Maxwell-Poisson Generalised Linear Models for egg abundance in all years (2006, 2007, 2008, 2020). Variables included are denoted with ‘+’. Ordered by AIC, with the optimal model in bold. All possible models are shown.

| Main effects | | | Interaction effects | |  |  |  |  |
| --- | --- | --- | --- | --- | --- | --- | --- | --- |
| Apparency | Year | Vegetation height | Year: Apparency | Year: Vegetation height | df | AIC | ΔAIC | Number of parameters |
| + | + | + | + | + | 12 | 1631.2 | 0 | 5 |
| **+** | **+** | **+** | **+** | **-** | **9** | **1632.1** | **0.87** | **4** |
| + | + | + | - | + | 9 | 1640.9 | 9.62 | 4 |
| + | + | + | - | - | 6 | 1640.9 | 9.67 | 3 |
| + | + | - | + | - | 8 | 1650.3 | 19.02 | 3 |
| + | + | - | - | - | 5 | 1660.9 | 29.68 | 2 |
| - | + | + | - | + | 8 | 1663.7 | 32.5 | 3 |
| - | + | + | - | - | 5 | 1665 | 33.76 | 2 |
| - | + | - | - | - | 4 | 1682.2 | 50.97 | 1 |
| + | - | + | - | - | 3 | 2783 | 1151.77 | 2 |
| + | - | - | - | - | 2 | 2837.3 | 1206.03 | 1 |
| - | - | + | - | - | 2 | 2968.1 | 1336.84 | 1 |
| - | - | - | - | - | 1 | 3040.9 | 1409.61 | 0 |

Table A6: Model selection table with Conway-Maxwell-Poisson Generalised Linear Models of egg abundance in 2007 and 2020 only, with main effects and interaction effects listed. Included variables are denoted with ‘+’. Ordered ascendingly by AIC, with the optimal model in bold. Only the top 50 models are shown.

| Main effects | | | | | | Interaction effects | | | | |  |  |  |  |
| --- | --- | --- | --- | --- | --- | --- | --- | --- | --- | --- | --- | --- | --- | --- |
| Apparency | Year | Buds | Solar radiation | Mature | Vegetation height | Year: Apparency | Year: Buds | Year: Solar radiation | Year: Mature | Year: Vegetation height | df | AIC | ΔAIC | Number of parameters |
| + | - | - | - | - | + | - | - | - | - | - | 3 | 936.386675 | 0 | 2 |
| **-** | **-** | **-** | **-** | **-** | **+** | **-** | **-** | **-** | **-** | **-** | **2** | **936.402172** | **0.0154967** | **1** |
| - | + | - | - | - | + | - | - | - | - | - | 3 | 937.468157 | 1.08148206 | 2 |
| + | + | - | - | - | + | - | - | - | - | - | 4 | 937.510584 | 1.12390853 | 3 |
| - | - | - | - | + | + | - | - | - | - | - | 3 | 937.667748 | 1.28107281 | 2 |
| + | - | - | - | + | + | - | - | - | - | - | 4 | 937.841835 | 1.45516011 | 3 |
| - | - | - | + | - | + | - | - | - | - | - | 3 | 938.035096 | 1.64842084 | 2 |
| + | - | + | - | - | + | - | - | - | - | - | 4 | 938.237485 | 1.85080995 | 3 |
| + | - | - | + | - | + | - | - | - | - | - | 4 | 938.292493 | 1.9058179 | 3 |
| - | - | + | - | - | + | - | - | - | - | - | 3 | 938.391302 | 2.0046265 | 2 |
| - | - | - | - | - | - | - | - | - | - | - | 1 | 938.695949 | 2.30927415 | 0 |
| - | + | - | + | - | + | - | - | - | - | - | 4 | 938.990361 | 2.60368569 | 3 |
| + | - | - | - | - | - | - | - | - | - | - | 2 | 939.08504 | 2.69836474 | 1 |
| - | - | - | + | + | + | - | - | - | - | - | 4 | 939.236635 | 2.8499593 | 3 |
| - | + | - | - | + | + | - | - | - | - | - | 4 | 939.264379 | 2.87770361 | 3 |
| + | + | - | + | - | + | - | - | - | - | - | 5 | 939.363277 | 2.97660135 | 4 |
| + | + | - | - | - | + | + | - | - | - | - | 5 | 939.374575 | 2.98789991 | 4 |
| + | + | - | - | + | + | - | - | - | - | - | 5 | 939.411433 | 3.02475786 | 4 |
| - | + | - | - | - | + | - | - | - | - | + | 4 | 939.451814 | 3.06513911 | 3 |
| - | + | + | - | - | + | - | - | - | - | - | 4 | 939.459031 | 3.0723559 | 3 |
| + | + | + | - | - | + | - | - | - | - | - | 5 | 939.473058 | 3.0863828 | 4 |
| + | + | - | - | - | + | - | - | - | - | + | 5 | 939.510094 | 3.12341869 | 4 |
| - | - | + | - | + | + | - | - | - | - | - | 4 | 939.650995 | 3.26431929 | 3 |
| + | - | + | - | + | + | - | - | - | - | - | 5 | 939.685152 | 3.29847709 | 4 |
| + | - | - | + | + | + | - | - | - | - | - | 5 | 939.709915 | 3.32323972 | 4 |
| - | - | + | + | - | + | - | - | - | - | - | 4 | 939.99834 | 3.61166438 | 3 |
| + | - | + | + | - | + | - | - | - | - | - | 5 | 940.110978 | 3.7243029 | 4 |
| - | + | - | - | - | - | - | - | - | - | - | 2 | 940.145629 | 3.75895374 | 1 |
| - | - | - | - | + | - | - | - | - | - | - | 2 | 940.487789 | 4.10111396 | 1 |
| + | + | - | - | - | - | - | - | - | - | - | 3 | 940.543538 | 4.15686254 | 2 |
| - | - | + | - | - | - | - | - | - | - | - | 2 | 940.634944 | 4.24826851 | 1 |
| - | - | - | + | - | - | - | - | - | - | - | 2 | 940.695673 | 4.30899753 | 1 |
| - | + | - | - | + | + | - | - | - | + | - | 5 | 940.717481 | 4.33080597 | 4 |
| - | + | - | + | + | + | - | - | - | - | - | 5 | 940.775088 | 4.38841229 | 4 |
| - | + | - | + | - | + | - | - | - | - | + | 5 | 940.928244 | 4.54156883 | 4 |
| + | - | - | - | + | - | - | - | - | - | - | 3 | 940.968477 | 4.58180215 | 2 |
| - | + | - | + | - | + | - | - | + | - | - | 5 | 940.979601 | 4.59292596 | 4 |
| - | + | + | + | - | + | - | - | - | - | - | 5 | 940.990326 | 4.60365067 | 4 |
| + | - | - | + | - | - | - | - | - | - | - | 3 | 940.994529 | 4.60785328 | 2 |
| + | - | + | - | - | - | - | - | - | - | - | 3 | 941.085012 | 4.69833646 | 2 |
| + | + | - | - | + | + | - | - | - | + | - | 6 | 941.158808 | 4.7721326 | 5 |
| - | - | + | + | + | + | - | - | - | - | - | 5 | 941.181943 | 4.79526742 | 4 |
| - | + | - | - | + | + | - | - | - | - | + | 5 | 941.229619 | 4.84294359 | 4 |
| + | + | - | + | + | + | - | - | - | - | - | 6 | 941.25374 | 4.86706482 | 5 |
| + | + | - | + | - | + | + | - | - | - | - | 6 | 941.26166 | 4.87498501 | 5 |
| - | + | + | - | + | + | - | - | - | - | - | 5 | 941.263704 | 4.87702863 | 4 |
| + | + | + | + | - | + | - | - | - | - | - | 6 | 941.307739 | 4.9210639 | 5 |
| + | + | - | - | + | + | + | - | - | - | - | 6 | 941.317341 | 4.93066601 | 5 |
| + | + | - | + | - | + | - | - | + | - | - | 6 | 941.325591 | 4.93891594 | 5 |
| + | + | + | - | - | + | + | - | - | - | - | 6 | 941.332083 | 4.94540722 | 5 |

**References**

Bedfordshire and Luton Biodiversity Recording and Monitoring Centre (2020). Retreived from: <https://www.bedscape.org.uk/BRMC/newsite/index.php?c=about_home>

Brereton TM, Botham MS, Middlebrook I, Randle Z, Noble D, Harris S, Dennis EB, Robinson A, Peck K and Roy DB (2020). United Kingdom Butterfly Monitoring Scheme report for 2019. UK Centre for Ecology & Hydrology, Butterfly Conservation, British Trust for Ornithology and Joint Nature Conservation Committee.

Met Office (2020) Met Office Integrated Data Archive System (MIDAS) Land and Marine Surface Stations Data (1853-current). NCAS British Atmospheric Data Centre, date of citation. <http://catalogue.ceda.ac.uk/uuid/220a65615218d5c9cc9e4785a3234bd0>
